# Serum creatinine/cystatin C ratio as a prognostic indicator for patients with colorectal cancer

**DOI:** 10.3389/fonc.2023.1155520

**Published:** 2023-06-20

**Authors:** Shunhui Gao, Hailun Xie, Lishuang Wei, Mingxiang Liu, Yanren Liang, Qiwen Wang, Shuangyi Tang, Jialiang Gan

**Affiliations:** ^1^ Department of Gastrointestinal Surgery, The Second People’s Hospital of Nanning, Nanning, China; ^2^ Guangxi Key Laboratory of Enhanced Recovery After Surgery for Gastrointestinal Cancer, The First Affiliated Hospital, Guangxi Medical University, Nanning, China; ^3^ Department of Colorectal and Anal Surgery, The First Affiliated Hospital, Guangxi Medical University, Nanning, China; ^4^ Department of Geriatric Respiratory Disease Ward, The First Affiliated Hospital, Guangxi Medical University, Nanning, China; ^5^ Department of Pharmacy, The First Affiliated Hospital, Guangxi Medical University, Nanning, China

**Keywords:** creatinine/cystatin C ratio, nutrition, colorectal cancer, progression-free survival, overall survival

## Abstract

**Background:**

This study aimed to explore the relationship between creatinine/cystatin C ratio and progression-free survival (PFS) and overall survival (OS) in colorectal cancer (CRC) patients undergoing surgical treatment.

**Methods:**

A retrospective analysis was conducted on 975 CRC patients who underwent surgical resection from January 2012 to 2015. Restricted three-sample curve to display the non-linear relationship between PFS/OS and creatinine-cystatin C ratio. Cox regression model and Kaplan-Meier method were used to evaluate the effect of the creatinine-cystatin C ratio on the survival of CRC patients. Prognostic variables with p-value ≤0.05 in multivariate analysis were used to construct prognostic nomograms. The receiver operator characteristic curve was used to compare the efficacy of prognostic nomograms and the traditional pathological stage.

**Results:**

There was a negative linear relationship between creatinine/cystatin C ratio and adverse PFS in CRC patients. Patients with low creatinine/cystatin C ratio had significantly lower PFS/OS than those with high creatinine/cystatin C ratio (PFS, 50.8% vs. 63.9%, p = 0.002; OS, 52.5% vs. 68.9%, p < 0.001). Multivariate analysis showed that low creatinine/cystatin C ratio was an independent risk factor for PFS (HR=1.286, 95%CI = 1.007–1.642, p=0.044) and OS (HR=1.410, 95%CI=1.087–1.829, p=0.010) of CRC patients. The creatinine/cystatin C ratio-based prognostic nomograms have good predictive performance, with a concordance index above 0.7, which can predict the 1–5-year prognosis.

**Conclusion:**

Creatinine/cystatin C ratio may be an effective prognostic marker for predicting PFS and OS in CRC patients, aid in pathological staging, and along with tumour markers help in-depth prognostic stratification in CRC patients.

## Introduction

Colorectal cancer (CRC) is the third and second most common cause of cancer morbidity and mortality, respectively, with nearly 2 million new cases and 1 million deaths reported worldwide in 2020 (1). CRC is the second most commonly diagnosed cancer and the fifth leading cause of cancer-related deaths in China (2). Surgical treatment remains the mainstay for CRC treatment. Social and economic development have led to increasingly improved methods for CRC treatment, including chemoradiotherapy, immunotherapy, traditional Chinese medicine treatment, and so on. Despite the progress in anti-tumour therapy for CRC, the long-term prognosis of CRC patients remains unsatisfactory, especially in patients with advanced CRC (3, 4). Therefore, it is necessary to find effective prognostic biomarkers to maximize the survival time of CRC patients.

At present, tumour-specific factors, such as pathological stage, perineural invasion, and vascular invasion, are the most commonly used tools for prognostic prediction, efficacy monitoring, and treatment formulation in CRC patients. However, due to their invasive nature, these tools have certain limitations. Additionally, owing to tumour heterogeneity, the prognosis of patients varies significantly within the same pathological stage (5, 6). Individual patient factors, including nutritional status, physical performance, and skeletal muscle mass, are also crucial to determine the prognosis of CRC patients. The detection and management of sarcopenia is a key aspect of the prognosis management in CRC patients. However, the diagnosis of sarcopenia requires device-dependent muscle mass measurements including dual-energy X-ray absorptiometry (DXA), computed tomography (CT), and bioelectrical impedance analysis (BIA), which are not widely used due to their high cost and radioactivity. Several studies have shown that straightforward, economical, and effective blood characteristics and their combinations can be used to accurately predict the clinical outcome of patients with cancer (7). Recent reports have suggested that the creatinine/cystatin C ratio in peripheral blood can be used to predict sarcopenia and prognosis in patients with cancer (8, 9). In 2017, Kashani et al. first developed the creatinine/cystatin C ratio and verified the correlation between creatinine/cystatin C ratio and sarcopenia (9). Since then, the creatinine/cystatin C ratio has been reported to be associated with sarcopenia and prognosis in various cancers (10–13). Serum creatinine and cystatin C are commonly used serum markers for evaluating glomerular filtration function in clinical practice (14). Serum creatinine is a derivative of the skeletal muscle protein creatine phosphate, which is mainly affected by the metabolism of muscle tissue *in vivo*; while, cystatin C, a small non-ionic protein derived exclusively from all nucleated cells and slightly metabolized by muscle tissue, may be used to estimate glomerular filtration function without concern for lean body mass and nutritional status (9, 15). Creatinine and cystatin C are derived from different cells. Creatinine reflects muscle metabolism, while cystatin C acts as a correction for renal function load. Therefore, the creatinine/cystatin C ratio may be a promising prognostic marker for CRC patients.

However, there is currently little research on the association between creatinine/cystatin C ratio and prognosis in CRC patients. Therefore, this study aimed to explore the relationship between creatinine/cystatin C ratio and progression-free survival (PFS) and overall survival (OS) in CRC patients undergoing surgical treatment, and develop a novel prognostic model based on creatinine/cystatin C ratio to accurately predict clinical outcomes.

## Patients and methods

### Study design

This study retrospectively included CRC patients who received surgical treatment in the Department of Colorectal and Anal Surgery, the First Affiliated Hospital, Guangxi Medical University from 2012 to 2015. The inclusion criteria were as follows: 1) patients diagnosed with CRC who received surgical treatment within a limited time; 2) serum creatinine, serum cystatin C, and other blood biochemical tests were performed within five days before surgery; 3) Patients with follow-up for at least 2 months; 4) Patients aged between 18 and 89 years old, with autonomy and no cognitive impairment. The exclusion criteria were as follows: 1) The primary site of the tumor is unclear or the tumor of multiple sources and multiple sites; 2) Patients with severe renal insufficiency or immune deficiency before surgery; 3) Patients who have received preoperative neoadjuvant radiotherapy or chemotherapy. This study strictly complied with the provisions of the Declaration of Helsinki and was approved by the Ethics Committee of the First Affiliated Hospital of Guangxi Medical University. Due to the retrospective study design, no informed consent was required.

### Data collection

Demographic and laboratory data were retrieved from electronic databases and patient medical records. Baseline demographic data included age, sex, height, weight, body mass index (BMI), and comorbidity (hypertension and diabetes). BMI is defined as body weight (kg)/height squared (m^2^). Pathological information included pathological stage, tumor stage (T stage), node stage (N stage), metastasis stage (M stage), perineural invasion, vascular invasion, pathological type, differentiation, tumor location, and maximum tumor size. Pathological staging adopted the TNM stage of the American Joint Committee on Cancer (AJCC) cancer staging 8th edition. Peripheral venous blood was collected from all patients after fasting for 8 hours in the week before surgery. Total blood count, serum creatinine concentration, serum cystatin C, and serum Carcinoembryonic antigen (CEA) were measured using an automated hematology analyzer (Beckman Coulter AU5800). The formula for calculating the serum creatinine/cystatin C ratio was as follows: (serum creatinine/serum cystatin C) × 100%.

### Follow-up and outcome

The CRC patients who recovered well after surgery were followed up regularly after discharge. Follow-up included regular visits to outpatient and inpatient clinics and telephone follow-up. The patients were followed up every 3 to 6 months in the first year and every 6 to 12 months starting in the second year until death. The main contents of the follow-up were inquiring about patients’ basic living conditions, serum tumor marker examination, abdominal CT, and electronic fiber colonoscopy. PFS was defined as the time interval between the date of the patient’s surgery and the patient’s disease recurrence or death. OS was defined as the time interval between the date of the patient’s surgery and death from any cause or the last follow-up. The last follow-up was on July 31, 2021.

### Statistical analysis

R language version 4.0.2 (http://www.R-project.org) statistical software was used for statistical analysis. Measurement data are expressed as mean ± standard deviation and were compared by an independent sample t-test. Enumeration data are expressed as numbers (percentages) and compared using chi-square tests. The optimal layering method was used to determine the optimal cutoff value of the creatinine-cystatin C ratio. Restricted Cubic Splines (RCS) are used to explore the associations between the creatinine-cystatin C ratio and PFS/OS. Survival curves were estimated by the Kaplan-Meier method, and survival rates were compared by the Log-rank test. Univariate and multivariate COX regression models were used to evaluate the risk factors affecting prognosis in CRC patients. Prognostic variables with p-value ≤0.05 in multivariate analysis were included to construct prognostic nomograms, and their discriminant ability was evaluated by the Concordance index (C-index). In addition, the calibration curves were used to compare the predicted probabilities of these nomograms with the actual results through 1000 resampling. The receiver operator characteristic curve (ROC) was used to compare the efficacy of prognostic nomograms and traditional TNM staging in predicting the prognosis of CRC patients. Finally, the total population was randomly divided into the validation cohorts at a ratio of 7:3 for internal validation. In this study, a two-tailed p-value less than 0.05 was considered statistically significant.

## Results

### Clinicopathological characteristics of the study population

According to inclusion criteria and exclusion criteria, 975 patients were finally eligible to be included in this study. Baseline clinicopathologic characteristics were shown in **Table 1**. The mean age of CRC patients was 57.50 ± 13.14 years old. 821 (63.0%) patients were men. There were 496 (48.1%) patients in the I-II stage and 479 (49.1%) patients in the III-IV stage. There were 476 cases (48.8%) of rectal cancer and 499 cases (51.2%) of colon cancer. Serum CEA was elevated (≥5.0 U/mL) in 411(42.2%) patients. Perineural invasion occurred in 88 (9.0%) patients and vascular invasion occurred in 145 (14.9%) patients. The optimal critical value of the creatinine-cystatin C ratio for predicting the prognosis of CRC patients was 106.75 (**Figure S1**). There were 734 CRC patients with a low creatinine/cystatin C ratio (<106.75) and 241 CRC patients with a high creatinine/cystatin C ratio (≥106.75). The median follow-up time of all patients was 72.8 months (38.9-88.4 months). A high creatinine/cystatin C ratio is significantly correlated with male gender, advanced age, low BMI, and prolonged hospital stay (**Table 2**).

**Table 1:** The clinicopathological factors of CRC patients.

Clinicopathological characteristics	Overall (n = 975)	Surviving patients (n = 551)	Deceased patients (n = 424)	p value
Sex(Man)	607 (62.3)	347 (63.0)	260 (61.3)	0.644
Age (mean (SD))	57.50 (13.14)	56.55 (12.19)	58.73 (14.20)	0.01
BMI (median [IQR])	57.50 (13.14)	56.55 (12.19)	58.73 (14.20)	0.01
Hypertension (Yes)	147 (15.1)	77 (14.0)	70 (16.5)	0.314
Diabetes (Yes)	61 (6.3)	30 (5.4)	31 (7.3)	0.289
T stage (T3-4)	735 (75.4)	369 (67.0)	366 (86.3)	<0.001
N stage				<0.001
N0	529 (54.3)	358 (65.0)	171 (40.3)	
N1	288 (29.5)	152 (27.6)	136 (32.1)	
N2	158 (16.2)	41 (7.4)	117 (27.6)	
M stage (Yes)	95 (9.7)	6 (1.1)	89 (21.0)	<0.001
TNM stage (III-IV)	479 (49.1)	199 (36.1)	280 (66.0)	<0.001
Perineural invasion (Yes)	88 (9.0)	32 (5.8)	56 (13.2)	<0.001
Vascular invasion (Yes)	145 (14.9)	50 (9.1)	95 (22.4)	<0.001
Macroscopic type				
Protrude type	243 (24.9)	156 (28.3)	87 (20.5)	0.018
Infiltrating type	89 (9.1)	46 (8.3)	43 (10.1)	
Ulcerative type	643 (65.9)	349 (63.3)	294 (69.3)	
Differentiation (Poor)	116 (11.9)	53 (9.6)	63 (14.9)	0.016
Tumor location (Rectal)	476 (48.8)	262 (47.5)	214 (50.5)	0.401
Tumor size (median [IQR])	4.50 (3.50, 6.00)	4.50 (3.50, 6.00)	5.00 (4.00, 6.00)	0.009
CEA (High)	411 (42.2)	187 (33.9)	224 (52.8)	<0.001
Creatinine	0.87 (0.74, 1.01)	0.87 (0.75, 1.01)	0.86 (0.71, 1.00)	0.328
Cystatin C	0.92 (0.80, 1.06)	0.91 (0.80, 1.03)	0.96 (0.82, 1.10)	0.002
Creatinine/cystatin C ratios	93.09(80.89,106.90)	94.39(82.32,110.44)	91.20(79.17,101.99)	<0.001
Radiotherapy (Yes)	65 (6.7)	40 (7.3)	25 (5.9)	0.474
Chemotherapy (Yes)	496 (50.9)	272 (49.4)	224 (52.8)	0.313
HOS (median [IQR])	12.00(10.00,14.00)	11.00 (9.00, 14.00)	12.00 (10.75, 15.00)	<0.001
Hospitalization cost (median [IQR])	49325.05(44483.04, 55547.88)	48503.38(44304.62, 54646.96)	50845.43(44607.71, 56887.80)	0.008

CRC, colorectal cancer; BMI, body mass index; CCR, creatinine/cystatin C ratio.

**Table 2:** The relationships between the CCR and clinicopathological factors of CRC patients.

Clinicopathological characteristics	CCR		p value
	Low (n = 734)	High (n = 241)	
Sex(Man)	400 (54.5)	207 (85.9)	<0.001
Age (mean (SD))	58.97 (13.25)	53.02 (11.74)	<0.001
BMI (median [IQR])	21.67 (19.60, 24.00)	22.32 (20.32, 24.56)	0.006
Hypertension (Yes)	114 (15.5)	33 (13.7)	0.556
Diabetes (Yes)	51 (6.9)	10 (4.1)	0.160
T stage (T3-4)	554 (75.5)	181 (75.1)	0.976
N stage			0.134
N0	402 (54.8)	127 (52.7)	
N1	206 (28.1)	82 (34.0)	
N2	126 (17.2)	32 (13.3)	
M stage (Yes)	74 (10.1)	21 (8.7)	0.62
TNM stage (III-IV)	358 (48.8)	121 (50.2)	0.755
Perineural invasion (Yes)	72 (9.8)	16 (6.6)	0.174
Vascular invasion (Yes)	106 (14.4)	39 (16.2)	0.579
Macroscopic type			0.874
Protrude type	182 (24.8)	61 (25.3)	
Infiltrating type	69 (9.4)	20 (8.3)	
Ulcerative type	483 (65.8)	160 (66.4)	
Differentiation (Poor)	93 (12.7)	23 (9.5)	0.236
Tumor location (Rectal)	364 (49.6)	112 (46.5)	0.444
Tumor size (median [IQR])	4.50 (3.50, 6.00)	4.50 (4.00, 6.00)	0.832
CEA (High)	318 (43.3)	93 (38.6)	0.224
Creatinine	0.81 (0.69, 0.94)	1.01 (0.89, 1.12)	<0.001
Cystatin C	0.96 (0.84, 1.10)	0.83 (0.75, 0.94)	<0.001
Creatinine/cystatin C ratios	87.31 (76.81, 95.49)	117.43 (111.88, 125.69)	<0.001
Death (Yes)	349 (47.5)	75 (31.1)	<0.001
Hospital stay (median [IQR])	12.00 (10.00, 14.00)	11.00 (9.00, 14.00)	0.006
Hospitalization cost (median [IQR])	49558.86 (44186.35, 55793.93)	48438.73 (44629.76, 54517.00)	0.260

CRC, colorectal cancer; BMI, body mass index; CCR, creatinine/cystatin C ratio.

### Kaplan-Meier survival curve of creatinine/cystatin C ratio for PFS

**Figure 1:**
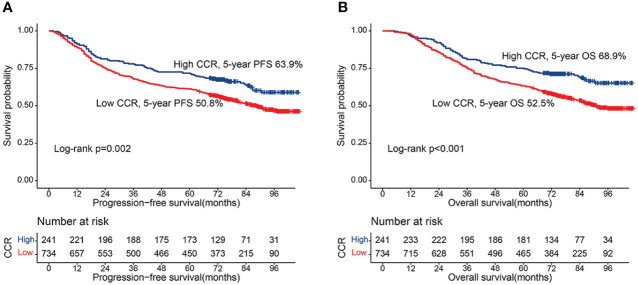
Kaplan-Meier curve of CCR in CRC patients. **(A)** Progression-free survival; **(B)** Overall survival.

**Figure 2:**
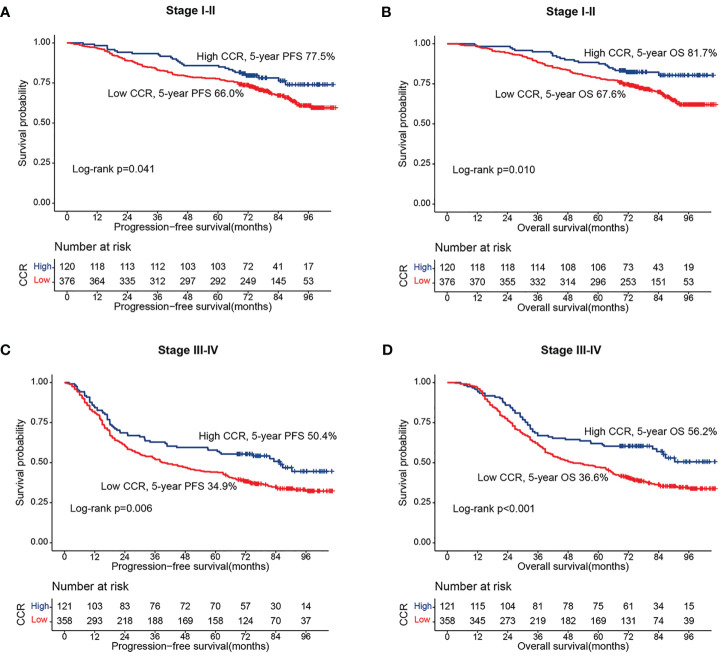
Stratified survival analysis of sarcopenia based on different TNM stage. **(A)** Progression-free survival of stage I-II; **(B)** Progression-free survival of stage III-IV; **(C)** Overall survival of stage I-II; **(D)** Overall survival of stage III-IV.

During follow-up, a total of 282 patients (28.9%) developed recurrence and metastasis, including 227 patients in the low creatinine/cystatin C ratio group (30.9%) and 55 patients in the high CCR group (22.8%). The 5-year RFS of patients in the low-creatinine/cystatin C ratio group was significantly lower than that in the high-creatinine/cystatin C ratio group (50.8% vs. 63.9%, p = 0.002) (**Figure 1A**). For stage I-II, PFS in patients with low creatinine/cystatin C ratio was significantly lower than that in patients with high creatinine/cystatin C ratio (66.0% vs 77.5%, p=0.041) (**Figure 2A**). For stage III-IV, we found that the creatinine/cystatin C ratio also significantly stratified the prognosis of CRC patients (34.9% vs 50.4%, p=0.006) (**Figure 2C**). In the normal CEA subgroup, we found that patients with low creatinine/cystatin C ratio had significantly lower 5-year PFS than those with high creatinine/cystatin C ratio (**Figure S2A**). However, no significant difference was observed in the high CEA subgroup (**Figure S2C**). The subgroup based on tumor location showed that the creatinine/cystatin C ratio could effectively stratify the prognosis of patients with rectal cancer (**Figure S3A**). Although the prognosis of rectal cancer with low creatinine/cystatin C ratio was worse than that of rectal cancer with high CCR, there was no significant difference (**Figure S3C**).

### Kaplan-Meier survival curve of creatinine/cystatin C ratio for OS

During the follow-up period, a total of 424 patients (43.5%) died, including 349 patients in the low creatinine/cystatin C ratio group (47.5%) and 75 patients in the low creatinine/cystatin C ratio group (31.1%). Kaplan-Meier survival curve showed that patients with low creatinine/cystatin C ratio had significantly lower OS than that with high creatinine/cystatin C ratio (52.5% vs. 68.9%, p < 0.001) (**Figure 1B**). In the TNM stage subgroup analysis, we found that CCR can effectively stratify the prognosis of CRC patients with stage I-II (67.6% vs 81.7%, p=0.010) (**Figure 2B**) and stage III-IV (36.6% vs 56.2%, p=0.010) (**Figure 2D**). Likewise, the creatinine/cystatin C ratio was able to significantly stratify the prognosis of patients with normal CEA (59.4% vs 79.1%, p<0.001) (**Figure S2B**), but not high CEA (**Figure S2D**). It is worth noting that the creatinine/cystatin C ratio can perform good prognostic differentiation on rectal cancer (50.8% vs 68.8%, p=0.003) and colon cancer (54.1% vs 69.0%, p=0.009) (**Figure S3B, D**).

### Multivariate analysis of predictors for PFS

**Figure 3:**
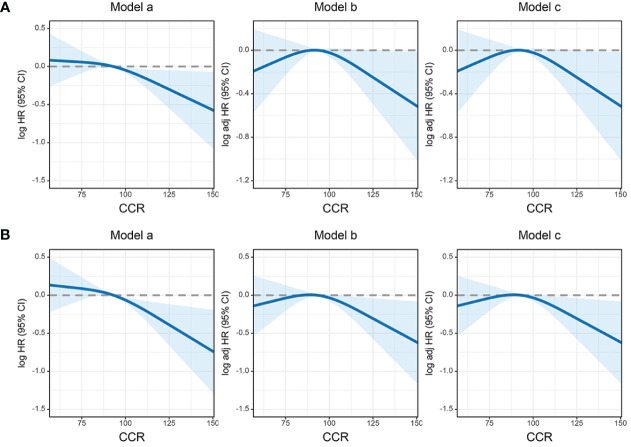
The association between CIPI and survival in patients with colorectal cancer. **(A)** Progression-free survival; **(B)** Overall survival. Model a: No adjusted. Model b: Adjusted for gender, age, and BMI. Model c: Adjusted for gender, age, BMI, hypertension, diabetes, T stage, N stage, M stage, tumor location, tumor size, perineural invasion, vascular invasion, macroscopic type, differentiation, radiotherapy, chemotherapy.

RCS showed that with the increase of creatinine/cystatin C ratio, The PFS of CRC patients gradually increased. After correcting for confounding factors, there was still a negative linear relationship between creatinine/cystatin C ratio and adverse PFS of CRC patients (**Figure 3**). In univariate analysis, PFS was affected by the following clinical characteristics: age (p=0.005), BMI, T stage (p<0.001), N stage (p<0.001), M stage (p<0.001), perineural invasion (p<0.001), vascular invasion (p<0.001), pathological type, differentiation (p=0.011), CEA (p<0.001) and creatinine/cystatin C ratio (p=0.002). Subsequent multivariate analysis of the 11 significant factors in the univariate analysis showed that the independent prognostic factors affecting PFS in CRC patients were age (HR=1.318, 95%CI=1.076–1.615, p=0.008), creatinine/cystatin C ratio (HR=1.286, 95%CI = 1.007–1.642, p=0.044), T stage (HR=1.559, 95%CI =1.173–2.073, p=0.002), N stage (p<0.001), M stage (HR=3.628, 95%CI=2.814–4.677, p<0.001) and CEA (HR=1.286, 95%CI=1.007–1.642, p=0.003) (**Table 3**). We performed a multivariate subgroup analysis based on various clinical features. The results showed that the low creatinine/cystatin C ratio was a risk factor for PFS in most of the subgroups (**Figure S4A**).

**Table 3:** Univariate and multivariate Cox regression analysis of clinicopathological characteristics associated with progression-free survival in CRC patients.

Clinicopathological characteristics	Progression-free survival			
	Univariate analysis		Multivariate analysis	
	HR (95%CI)	P value	HR (95%CI)	P value
Age	1.315 (1.085-1.594)	0.005	1.318 (1.076 - 1.615)	0.008
BMI				
Low	Ref.		Ref.	
Normal	0.843 (0.651-1.091)	0.194	0.904 (0.695 - 1.175)	0.450
High	0.673 (0.498-0.909)	0.010	0.799 (0.588 - 1.084)	0.149
T stage (T3/4)	2.48 (1.9-3.238)	<0.001	1.559 (1.173 - 2.073)	0.002
N stage				
N0	Ref.		Ref.	
N1	1.724 (1.385-2.145)	<0.001	1.457 (1.16 - 1.83)	0.001
N2	3.754 (2.978-4.733)	<0.001	2.692 (2.096 - 3.459)	<0.001
M stage	5.693 (4.483-7.228)	<0.001	3.628 (2.814 - 4.677)	<0.001
Perineural invasion (Positive)	1.788 (1.36-2.352)	<0.001	1.094 (0.806 - 1.483)	0.565
Vascular invasion (Positive)	1.937 (1.547-2.427)	<0.001	1.284 (0.991 - 1.662)	0.058
Pathological type				
Protrude type	Ref.		Ref.	
Infiltrating type	1.447 (1.017-2.061)	0.04	1.196 (0.835 - 1.713)	0.328
Ulcerative type	1.29 (1.023-1.626)	0.032	1.077 (0.85 - 1.365)	0.537
Differentiation (High-medium)	0.707 (0.541-0.923)	0.011	0.901 (0.681 - 1.193)	0.468
CEA (≥5ng/ml)	1.919 (1.593-2.31)	<0.001	1.358 (1.111 - 1.66)	0.003
CCR (Low)	1.456 (1.152-1.84)	0.002	1.286 (1.007 - 1.642)	0.044

CRC, colorectal cancer; BMI, body mass index; CCR, creatinine/cystatin C ratio.

### Multivariate analysis of predictors for OS

In univariate Cox proportional hazard regression models, patients with low creatinine/cystatin C ratio had 1.410 times the risk of adverse OS compared with patients with high creatinine/cystatin C ratio (HR = 1.421, 95%CI =1.168 – 1.730, p<0.001). After adjusting for confounding factors, advanced age (HR=1.013, 95%CI=1.005–1.021, p=0.001), low creatinine/cystatin C ratio (HR=1.410, 95%CI=1.087–1.829, p=0.010), advanced T stage (HR=1.578, 95%CI=1.175–2.120, p=0.002), advanced N stage (p<0.001), advanced M stage (HR=3.879, 95%CI=3.001–5.013, p<0.001) and high CEA (HR=1.333, 95%CI=1.084–1.641, p=0.007) were independently associated with poor OS in CRC patients (**Table 4**). Multivariate subgroup analysis showed that a low creatinine/cystatin C ratio was a risk factor for OS in most subgroups of CRC patients (**Figure S4B**).

**Table 4:** Univariate and multivariate Cox regression analysis of clinicopathological characteristics associated with overall survival in CRC patients.

Clinicopathological characteristics	Overall survival			
	Univariate analysis		Multivariate analysis	
	HR (95%CI)	P value	HR (95%CI)	P value
Age	1.421 (1.168-1.73)	<0.001	1.013 (1.005 - 1.021)	0.001
BMI				
Low	Ref.			
Normal	0.846 (0.648-1.102)	0.215	0.920 (0.702 - 1.207)	0.548
High	0.661 (0.484-0.902)	0.009	0.787 (0.573 - 1.079)	0.137
T stage (T3/4)	2.52 (1.91-3.326)	<0.001	1.578 (1.175 - 2.120)	0.002
N stage				
N0	Ref.			
N1	1.7 (1.357-2.129)	<0.001	1.451 (1.146 - 1.837)	0.002
N2	3.77 (2.974-4.779)	<0.001	2.633 (2.034 - 3.409)	<0.001
M stage	6.096 (4.792-7.755)	<0.001	3.879 (3.001 - 5.013)	<0.001
Perineural invasion (Positive)	1.782 (1.345-2.36)	<0.001	1.052 (0.768 - 1.442)	0.750
Vascular invasion (Positive)	2.019 (1.606-2.538)	<0.001	1.335 (1.024 - 1.74)	0.033
Pathological type				
Protrude type				
Infiltrating type	1.425 (0.989-2.053)	0.058	1.208 (0.831 - 1.756)	0.322
Ulcerative type	1.295 (1.02-1.646)	0.034	1.118 (0.873 - 1.43)	0.377
Differentiation (High-medium)	0.648 (0.496-0.847)	0.001	0.802 (0.602 - 1.07)	0.134
Size (≥5cm)	1.275 (1.053-1.543)	0.013	1.061 (0.869 - 1.296)	0.563
CEA (≥5ng/ml)	1.896 (1.566-2.295)	<0.001	1.333 (1.084 - 1.641)	0.007
CCR (Low)	1.646 (1.283-2.113)	<0.001	1.410 (1.087 - 1.829)	0.010

CRC, colorectal cancer; BMI, body mass index; CCR, creatinine/cystatin C ratio.

### Construction of prognosis prediction model

**Figure 4:**
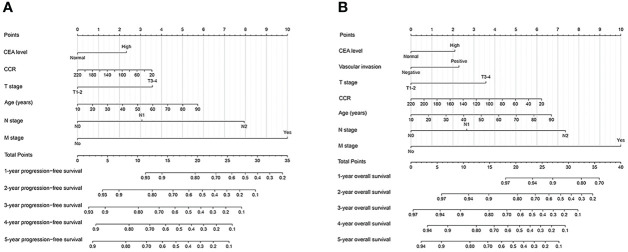
Construction prognostic nomograms in CRC patients. **(A)** The Progression-free survival nomogram; **(B)** The overall survival nomogram.

To evaluate the prognosis of CRC patients comprehensively, we have built prognostic nomograms for predicting the prognosis of 1-5 years of CRC patients. Based on all independent indicators in the multivariate analysis of PFS, we constructed a PFS nomogram, including age, T stage, N stage, M stage, CEA levels, and creatinine/cystatin C ratio (**Figure 4A**). The higher the nomogram score, the worse the clinical prognosis of CRC patients. The C-index of the PFS nomogram was 0.719 (95%CI: 0.695-0.743). The 3 - and 5-year calibration curves showed good agreement between the predicted values of the nomogram and observed values (**Figures S5A, B**). Similarly, we included significant variables in the multivariate analysis of OS to construct an OS nomogram (including age, T stage, N stage, M stage, vascular invasion, CEA level, and creatinine/cystatin C ratio) (**Figure 4B**). The C-index of the OS nomogram was 0.727 (95%CI: 0.703-0.752). The 3 -year and 5 -year calibration curves demonstrated the best agreement between the survival probability predicted by the OS nomogram and the actual observed values (**Figures S5C, D**). Subsequently, we compared the constructed nomograms with the traditional TNM staging through the ROC curve. Compared with TNM staging, the constructed nomograms had better resolution and accuracy in predicting 3 - and 5-year PFS (3-year AUC: 0.773 vs 0.734; 5-year AUC: 0.767 vs 0.720) (**Figures S6A, B**). Similarly, the constructed nomograms were better at predicting the performance of the 3 - and 5-year OS than the TNM staging (3-year AUC: 0.782 vs 0.742; 5-year AUC: 0.772 vs 0.718) (**Figures S6C, D**).

### Internal queue validation

**Figure 5:**
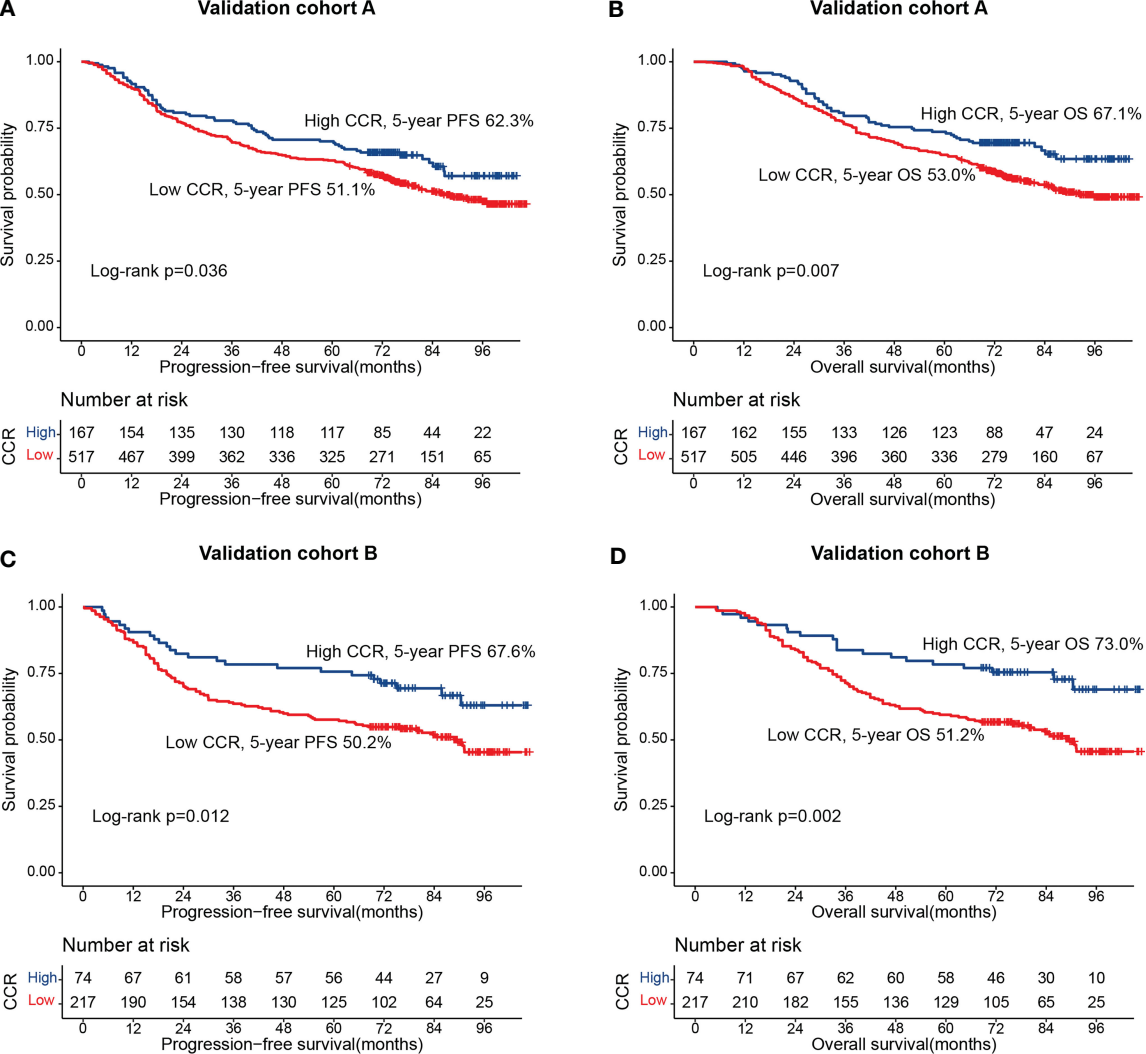
The association between CCR and survival in patients with colorectal cancer in validation cohorts. **(A)** Progression-free survival at validation cohort A; **(B)** Overall survival at validation cohort A; **(C)** Progression-free survival at validation cohort B; **(D)** Overall survival at validation cohort B.

We randomly divide all patients into two cohorts at a ratio of 7: 3: validation cohort A (684 cases) and validation cohort B (291 cases). **Table S1** showed that there was no statistical significance in clinicopathological characteristics between the validation cohort A group and validation cohort B group. In the validation cohort, A, patients in the low-creatinine/cystatin C ratio group had significantly lower PFS (51.1% vs. 62.3%, p = 0.036) (**Figure 5A**) and OS (53.0% vs. 67.1%, p = 0.007) (**Figure 5B**) than those in the high-creatinine/cystatin C ratio group. In validation cohort B, the creatinine/cystatin C ratio was still able to effectively stratify the prognosis of CRC patients (PFS, 50.2% vs. 67.6%, p = 0.012; OS, 51.2% vs. 73.0%, p = 0.002) (**Figures 5C, D**). The C-index of PFS and OS nomograms was 0.715 (95%CI: 0.687-0.743) and 0.726 (95%CI: 0.698-0.754) at validation cohort A, respectively. In validation cohort B, The C-index of PFS and OS nomograms was 0.742 (95%CI: 0.700-0.785) and 0.740 (95%CI: 0.696-0.785), respectively. In addition, calibration curves of 3- and 5-year PFS/OS in validation cohort A (**Figures S7A, B**) and validation cohort B (**Figures S7C, D**) showed good agreement between predicted and observed values.

## Discussion

In this study, we demonstrated for the first time that the creatinine/cystatin C ratio is an important predictor of PFS and OS in CRC patients. With the increase of creatine-cystatin C ratio, the HRS of mortality of CRC patients gradually decreases. The creatinine/cystatin C ratio can also be used as an effective auxiliary tool for pathological staging to further distinguish the prognosis of CRC patients with the same pathological stage. We also found that CCR could further stratify the prognosis of CRC patients with normal CEA, but was not suitable for patients with high CEA. In addition, we constructed CCR-based prognostic nomograms to predict 1-5year PFS/OS in CRC patients and validated the good predictive performance of these nomograms through the random internal cohorts.

At present, the relationship between the creatinine/cystatin C ratio and the prognosis of patients with cancer has attracted more and more attention. Jung et al. (16) found that the creatinine-cystatin C ratio was significantly associated with reduced 6-month mortality of patients with cancer. Ding et al. (17) also found that the creatinine/cystatin C ratio was independently correlated with sarcopenia and relapse-free survival in patients with gastrointestinal stromal tumors. The study of Zheng et al. (11) also showed that creatinine/cystatin C ratio can be used to identify sarcopenia and is a useful prognostic factor for postoperative complications and long-term survival in patients with esophageal cancer. A study by Chen et al. involving 664 non-small cell lung cancer patients found that creatinine/cystatin C ratio was associated with mortality in women, but not in men (18). The results of this study showed that CRC patients in the high creatinine/cystatin C ratio had significantly higher RFS/OS than those in the low serum creatinine/cystatin C ratio group. Multivariate analysis showed that CRC patients with low creatinine/cystatin C ratio had 28.6% and 41.0% higher adverse PFS and OS than CRC patients with high creatinine/cystatin C ratio, respectively.

Pathological stages and serum CEA levels are important factors in assessing the prognosis of CRC patients (5). However, even with the same pathological stage, the prognosis of patients varies greatly. In this study, we found that the creatinine/cystatin C ratio can effectively stratify the prognosis of CRC patients with the same level of pathological staging, suggesting that the creatinine/cystatin C ratio can be a useful supplement in predicting the prognosis of CRC patients. Studies have shown that serum CEA is not specific in CRC patients, and more than 50%of CRC patients have negative serum CEA (19, 20). We found that the creatinine/cystatin C ratio can be used as an effective prognostic stratification factor for CRC patients with normal CEA levels, suggesting that it can be used as a further prognostic stratification factor for CEA in the prognosis prediction of CRC patients. Furthermore, the high creatinine/cystatin C ratio was a prognostic factor for poor PFS/OS in patients with colon cancer. However, the creatinine/cystatin C ratio is a useful prognostic factor in predicting OS in rectal cancer patients, but not PFS. Overall, the creatinine/cystatin C ratio can be considered a universally applicable, readily available, and effective method for predicting the risk of poor outcomes in CRC patients.

One possible explanation for the association between the creatinine-cystatin C ratio and the outcome of CRC patients is that the creatinine-cystatin C ratio represents muscle mass, which is a well-known risk factor for the outcome of CRC patients (21). A recent study showed that the creatinine/cystatin C ratio was significantly correlated with CT and BIA in assessing muscle mass, and could be conveniently used as a reliable biomarker for muscle in patients with cancer (22). Similarly, a study by Tlemsani et al. also showed that the creatinine/cystatin C ratio is a useful and simple biomarker for predicting sarcopenia in patients with cancer. In addition, the index also appears to be a strong biomarker for the diagnosis of sarcopenia in overweight and obese cancer patients (11). These studies further support this explanation. Other research suggested that the creatinine/cystatin C ratio may also be a sign of systemic inflammation (16, 23). Systemic inflammation is the most representative interaction between tumor and host, and increased inflammation load is an important factor affecting the prognosis of cancer patients (7). Serum creatinine levels were lower in patients with high white blood cell counts, while elevated levels of cystatin C were observed in chronic inflammatory states. The decrease in the creatinine/cystatin C ratio reflects the accumulation of inflammatory load in the body. Therefore, the creatinine/cystatin C ratio may be a promising prognostic biomarker in CRC patients.

Further, we constructed creatinine/cystatin C ratio-based prognostic nomograms to predict the 1–5-year prognosis of CRC patients. These nomograms have good predictive performance, with C-index can reach above 0.7. Subsequently, we demonstrated that these nomograms had good application prospects through validation cohorts. Compared to traditional pathological stages, these nomograms have better prognostic prediction efficiency. In summary, these nomograms have good prognostic efficacy, which can help to provide individualized recommendations for prognostic prediction, efficacy evaluation, and treatment formulation of CRC patients.

As far as we know, this study is the first to confirm that the creatinine/cystatin C ratio is an independent predictor of PFS and OS in CRC patients. Creatinine/cystatin C ratio also can help pathological staging and tumor markers to stratify the prognosis of CRC patients in more detail. In addition, we further constructed the prognostic nomograms based on the creatinine/cystatin C ratio, which can be more personalized and convenient to be used in clinical practice. However, there are still some limitations worth noting. This is a single-center retrospective study, with problems such as small sample size and patient selection bias. Secondly, this study lacks data to evaluate sarcopenias, such as CT, DXA, and BIA, which further restricts the explanation of the association between creatinine/cystatin C ratio and muscle mass. As patients can experience kidney damage due to surgery, radiation therapy, and chemotherapy, resulting in fluctuations in creatinine and cystatin C levels, peripheral venous blood samples that include Creatinine and Cystatin C were collected from all patients after an overnight fast during the week before surgery in this study. However, as this study only collected single-time serum data, we were unable to explore the impact of the trajectory changes of Creatinine/cystatin C ratio on prognosis. Finally, this study lacks an independent validation cohort, which is an additional limitation. Therefore, further prospective studies with multi-center and large sample sizes are needed.

## Conclusion

Creatinine/cystatin C ratio may be an effective prognostic marker for predicting PFS and OS in CRC patients and can help pathological staging and tumor markers to perform more detailed prognostic stratification in CRC patients. The creatinine/cystatin C ratio-based nomograms have good prediction accuracy and can individually help identify high-risk patients who have an adverse prognosis.

## Data availability statement

The original contributions presented in the study are included in the article/**Supplementary Material**. Further inquiries can be directed to the corresponding authors.

## Ethics statement

The studies involving human participants were reviewed and approved by the Institutional Review Board of the First Affiliated Hospital, Guangxi Medical University. The patients/participants provided their written informed consent to participate in this study.

## Author contributions

JG, SG, and HX carried out the design of this study, analyses of statistics and draft the manuscript. HX, LW, ML, YL, QW, and ST carried out collection of the statistics and prepared the manuscript. All authors contributed to the article and approved the submitted version.
