# Supplementary material for: Serum creatinine/cystatin C ratio as a prognostic indicator for patients with colorectal cancer
Source: Front Oncol. 2023 Jun 20;13:1155520. doi: 10.3389/fonc.2023.1155520 (PMC10319047; doi:10.3389/fonc.2023.1155520)
Supplement: Supplementary file 1 [file DataSheet_1.docx]

**Figure S1.** The optimal threshold of CCR in patients with colorectal cancer.

**
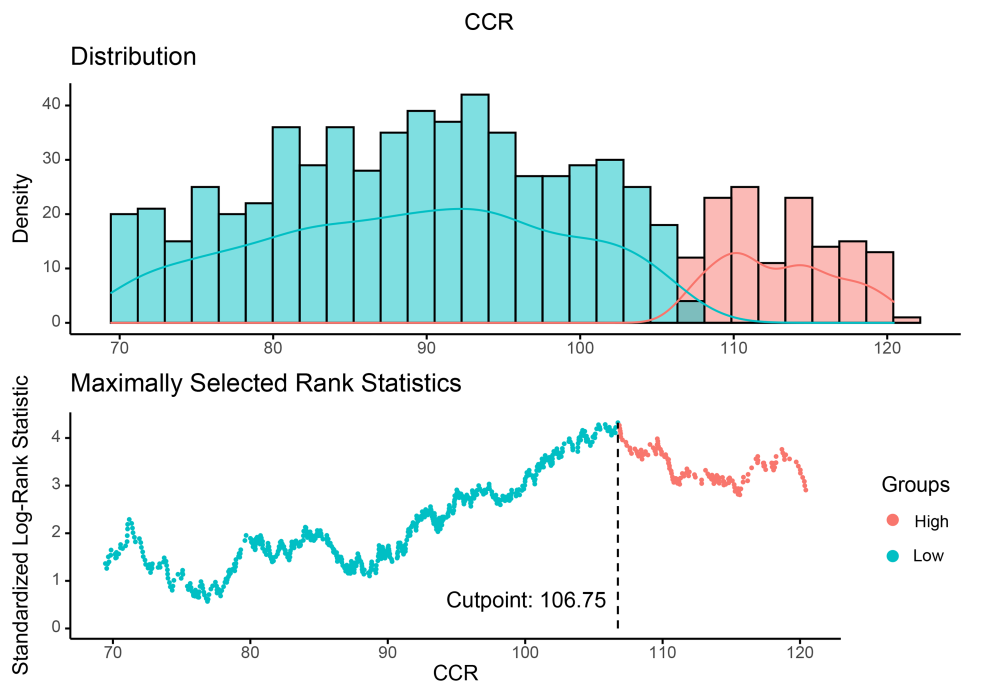
**

**Figure S2.** Stratified survival analysis based on CEA level.


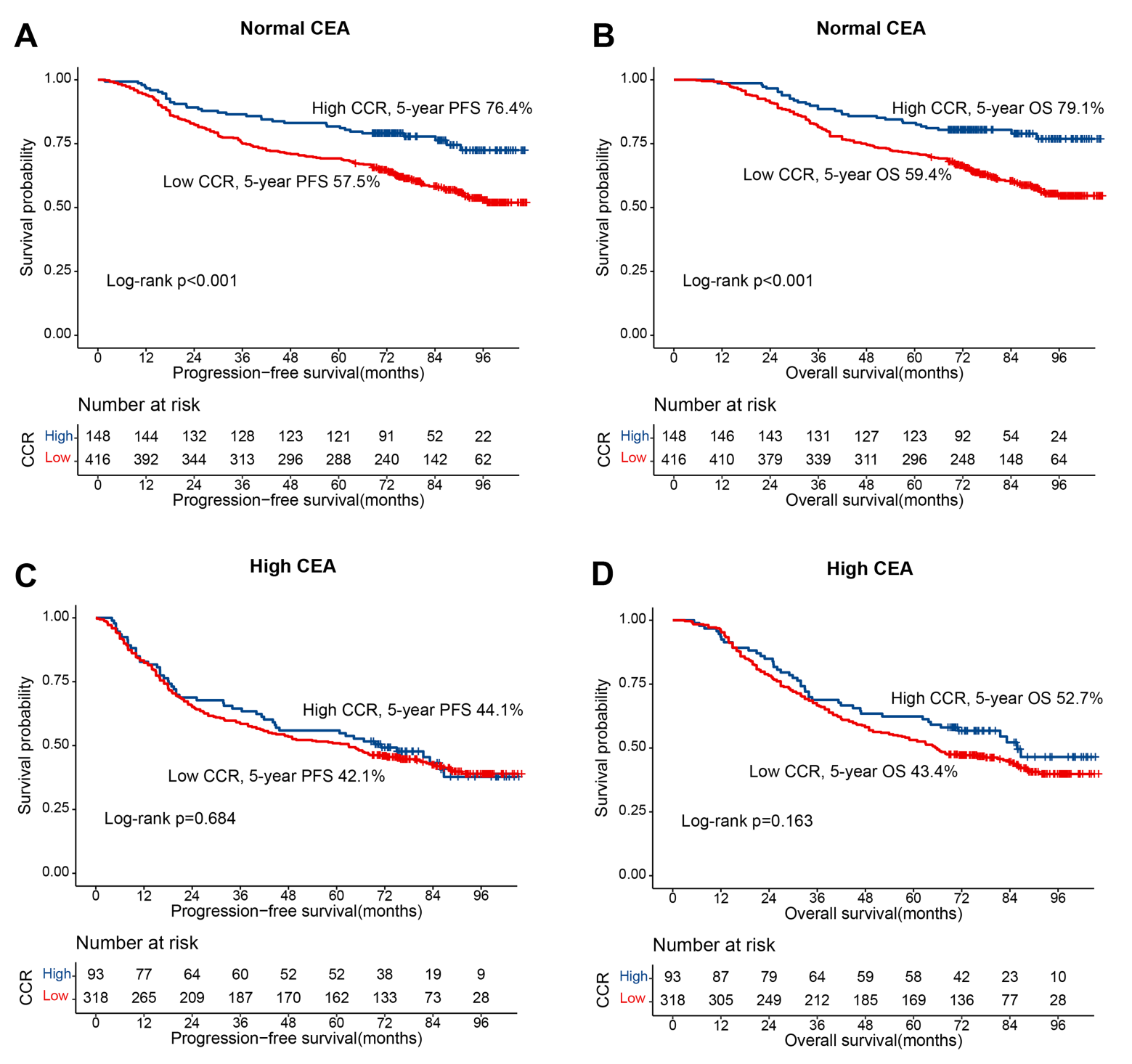


Notes: A, Progression-free survival of sarcopenia at normal CEA level; B, Overall survival of sarcopenia at normal CEA level; C, Progression-free survival of PINI at high CEA level; D, Overall survival of Progression at high CEA level.

**Figure S3.** Stratified survival analysis based on tumor location.


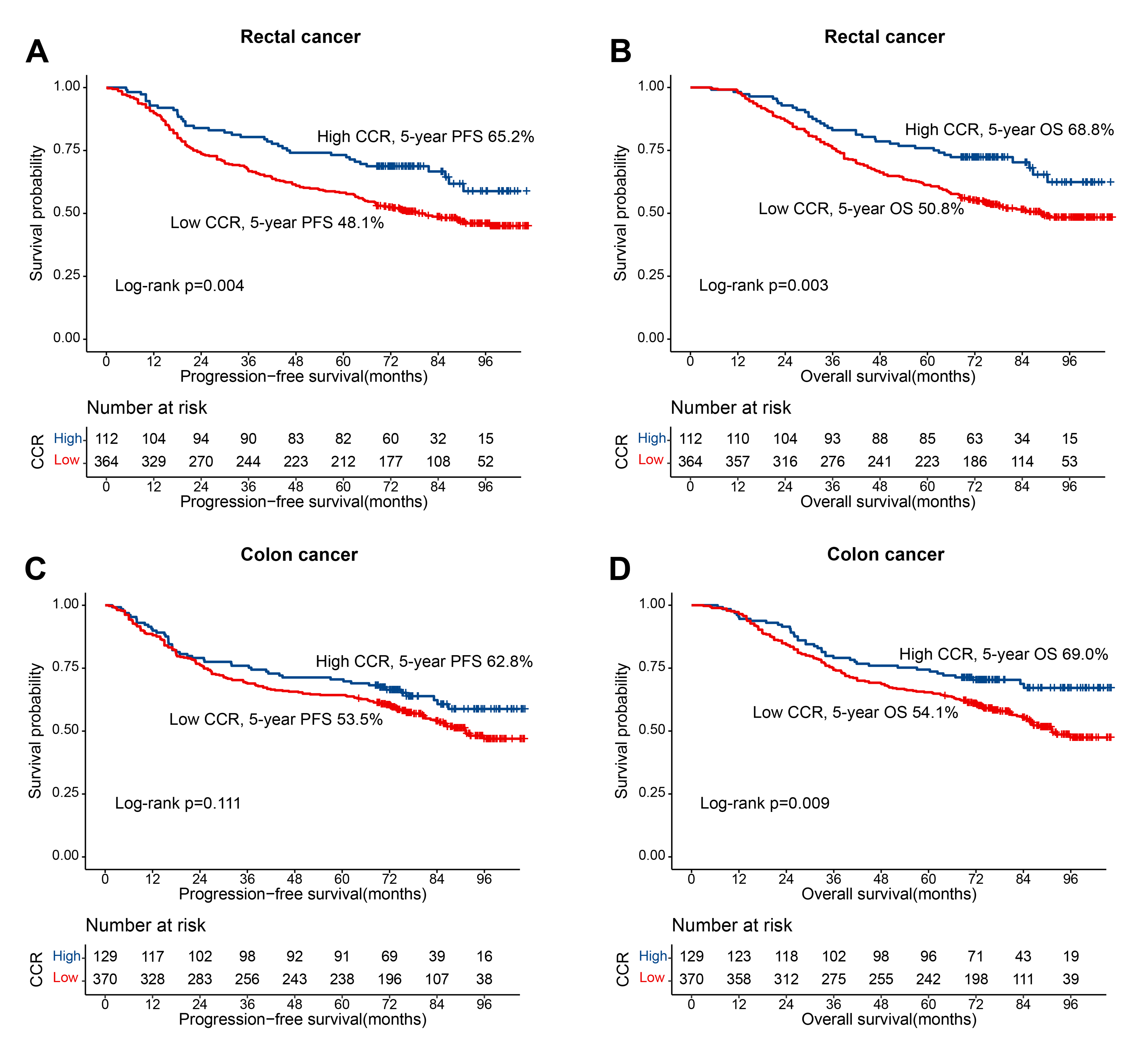


Notes: A, Progression-free survival of sarcopenia at normal CEA level; B, Overall survival of sarcopenia at normal CEA level; C, Progression-free survival of PINI at high CEA level; D, Overall survival of Progression at high CEA level.

**Figure S4.** The association between CCR and hazard risk of survival in various subgroups. (A, Progression-free survival, B, Overall survival).


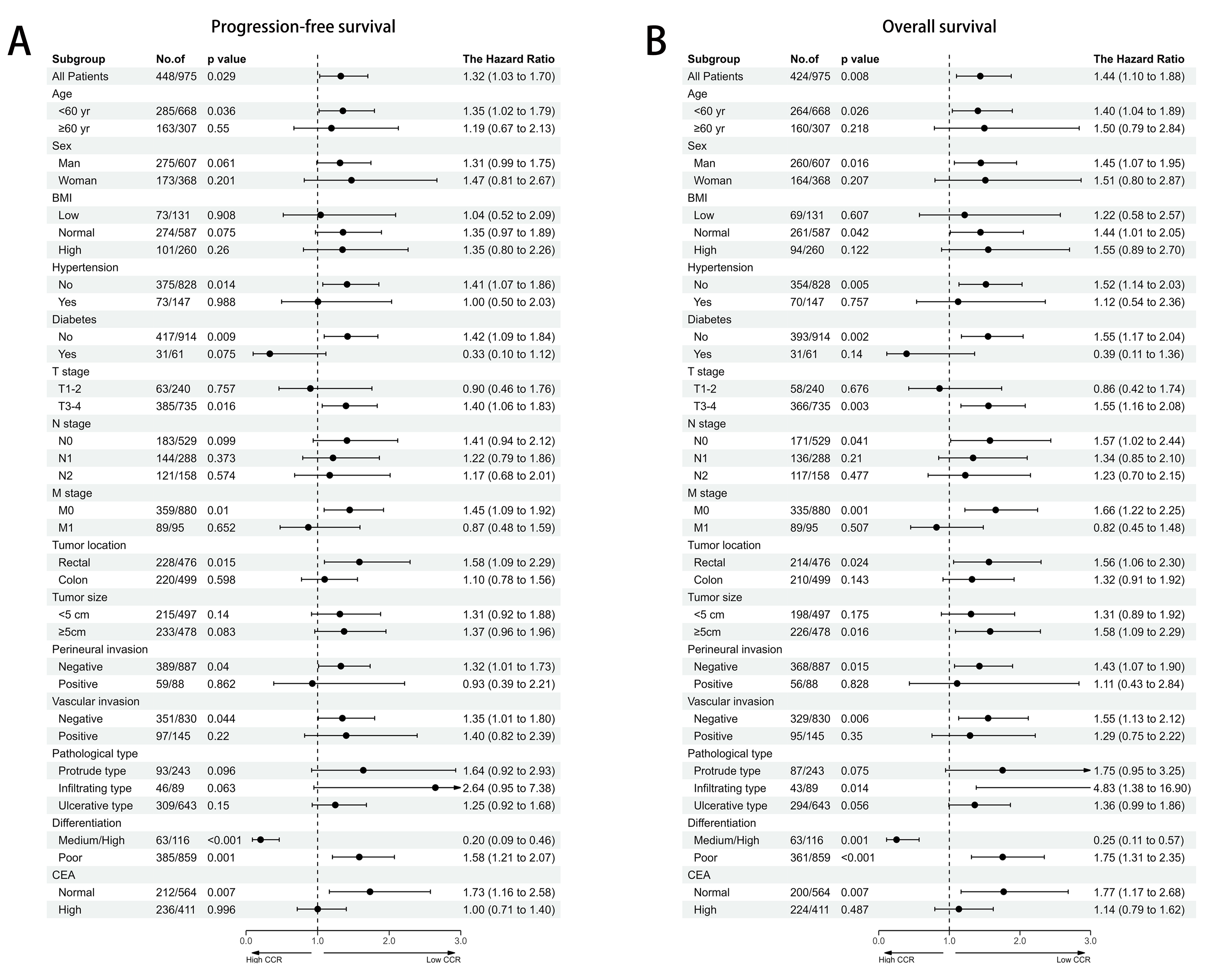


**Figure S5.** Calibration curve of the prognostic nomograms.

**
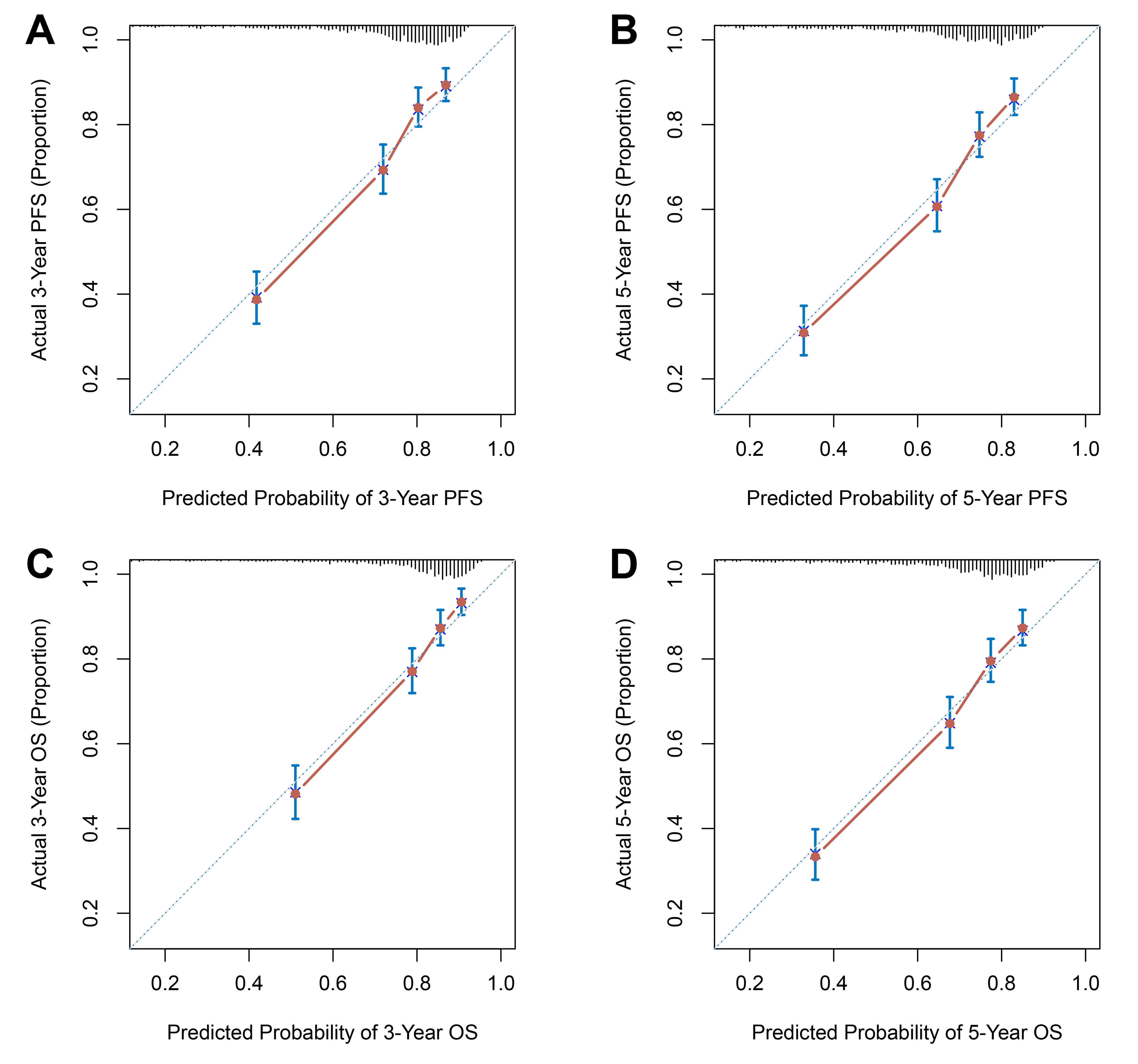
**

Notes: A, 3-year progression-free survival; B, 5-year progression-free survival; C, 3-year overall survival; D, 5-year overall survival.

**Figure S6.** Comparison of the ability of the novel prognostic nomograms and TNM stage in predicting PFS and OS of patients with colorectal cancer.

**
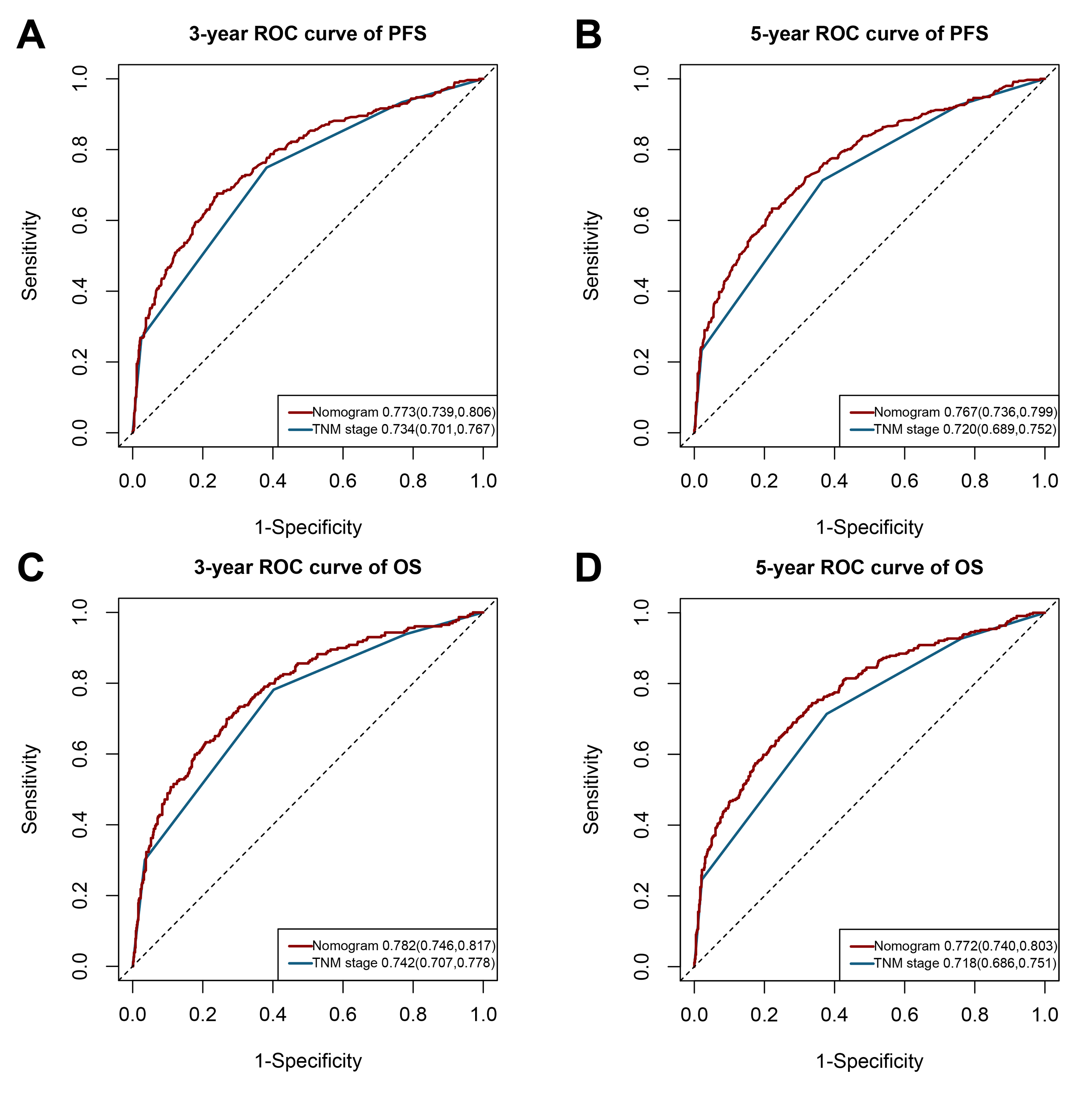
**

Notes: A, 3-year DFS; B, 5-year DFS; C, 3-year OS; D, 5-year OS.

**Figure S7.** Calibration curve at randomize internal validations.

**
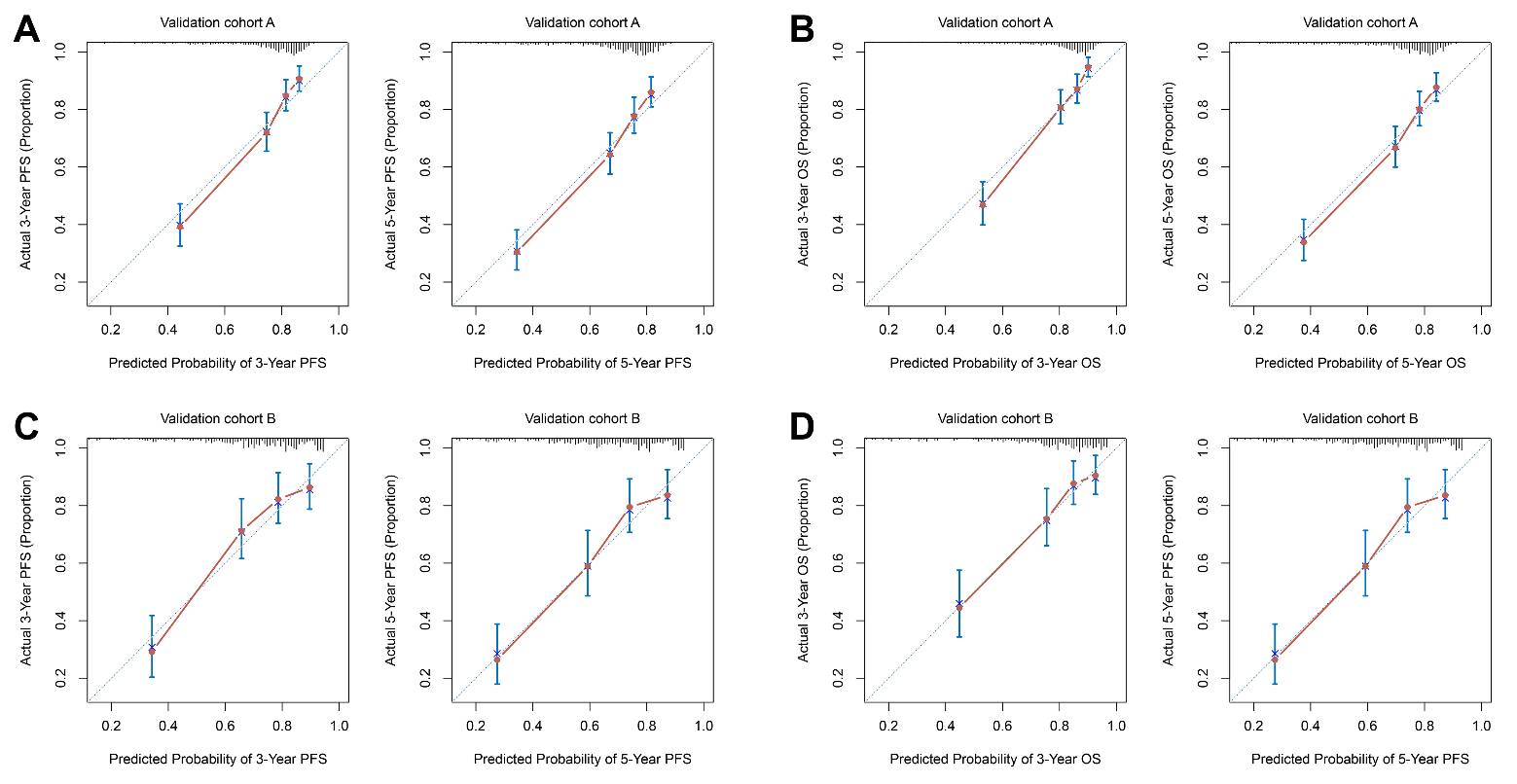
**

**Notes:** A, Validation cohort A; B, Validation cohort B.

**Table S1.** The clinicopathological Features of two validation cohorts in CRC patients.

| Clinicopathological characteristics | Validation cohort A  (n = 684) | Validation cohort B  (n = 291) | P value |
| --- | --- | --- | --- |
| Sex(Man) | 424 (62.0) | 183 (62.9) | 0.847 |
| Age (mean (SD)) | 57.90 (13.00) | 56.55 (13.44) | 0.141 |
| BMI (median [IQR]) | 21.78 (19.83, 24.01) | 22.15 (19.82, 24.46) | 0.338 |
| Hypertension (Yes) | 105 (15.4) | 42 (14.4) | 0.788 |
| Diabetes (Yes) | 39 ( 5.7) | 22 ( 7.6) | 0.341 |
| T stage (T3/4) | 507 (74.1) | 228 (78.4) | 0.186 |
| N stage |  |  | 0.244 |
| N0 | 377 (55.1) | 152 (52.2) |  |
| N1 | 205 (30.0) | 83 (28.5) |  |
| N2 | 102 (14.9) | 56 (19.2) |  |
| M stage (Yes) | 66 ( 9.6) | 29 (10.0) | 0.972 |
| TNM stage (III-IV) | 329 (48.1) | 150 (51.5) | 0.36 |
| Perineural invasion (Yes) | 69 (10.1) | 19 ( 6.5) | 0.098 |
| Vascular invasion (Yes) | 106 (15.5) | 39 (13.4) | 0.458 |
| Macroscopic type |  |  | 0.702 |
| Protrude type | 172 (25.1) | 71 (24.4) |  |
| Infiltrating type | 59 ( 8.6) | 30 (10.3) |  |
| Ulcerative type | 453 (66.2) | 190 (65.3) |  |
| Differentiation (Poor) | 82 (12.0) | 34 (11.7) | 0.979 |
| Tumor location (Rectal) | 342 (50.0) | 134 (46.0) | 0.289 |
| Tumor size (median [IQR]) | 4.50 (3.50, 6.00) | 5.00 (4.00, 6.00) | 0.224 |
| CEA (High) | 293 (42.8) | 118 (40.5) | 0.555 |
| Radiotherapy (Yes) | 54 ( 7.9) | 11 ( 3.8) | 0.027 |
| Chemotherapy (Yes) | 338 (49.4) | 158 (54.3) | 0.185 |
| Death (Yes) | 298 (43.6) | 126 (43.3) | 0.995 |
| HOS (median [IQR]) | 12.00 (9.75, 14.00) | 12.00 (10.00, 14.00) | 0.583 |
| Hospitalization cost (median [IQR]) | 49198.56 (44285.28, 55271.94) | 49894.09 (44607.79, 56536.70) | 0.233 |

Table Note: CRC, colorectal cancer; BMI, body mass index.
